# NUMBER OF LIVING SIBLINGS AND COGNITIVE DECLINE AMONG OLDER MEXICAN ADULTS

**DOI:** 10.1093/geroni/igae098.1064

**Published:** 2024-12-31

**Authors:** Nekehia Quashie, Joseph Saenz, Xing Zhang

**Affiliations:** University of Rhode Island, University of Rhode Island, Rhode Island, United States; Arizona State University, Phoenix, Arizona, United States; Arizona State University, Phoenix, Arizona, United States

## Abstract

**Objectives:**

A growing body of research examines the associations between sibship size and health in older adults. These studies largely focus on older adults’ physical health or mortality in high-income countries, yet cognitive health is understudied. Sibship size may impact cognitive development in early childhood and shapes opportunities for social support throughout life that potentially influence later life cognitive health. Using a gendered life course framework, this study evaluates how the number of living siblings in later life relates with cognitive health among older Mexican adults.

**Methods:**

Data comes from the 2012, 2015, 2018, and 2021 waves of the Mexican Health and Aging Study (n=14,872 adults age 50+). We use latent growth curve models to evaluate how one’s number of living siblings relates with levels of latent general cognitive ability and nine-year cognitive decline and how these associations may differ by gender.

**Results:**

Among men and women, those with no living siblings or only one living sibling had lower levels of cognitive ability when compared to those with 3 living siblings. However, very large sibship size (having 10+ living siblings) was also related with lower cognitive ability in men. Regardless of gender, the number of living siblings was not related with the rate of cognitive decline.

**Discussion:**

This research broadens our understanding of the salience of siblings, an overlooked family resource, to older adults’ health. Our findings underscore the importance of alternative social support for older adults given Mexico’s changing demographics that challenge the reliance on family support in late-life.

